# Sustainable Food Systems in Fruits and Vegetables Food Supply Chains

**DOI:** 10.3389/fnut.2022.829061

**Published:** 2022-02-17

**Authors:** Lucía Cassani, Andrea Gomez-Zavaglia

**Affiliations:** ^1^Instituto de Investigaciones en Ciencia y Tecnología de Materiales (INTEMA, CONICET), Mar del Plata, Argentina; ^2^Departamento de Ingeniería Química y en Alimentos, Facultad de Ingeniería, Universidad Nacional de Mar del Plata (UNMdP), Mar del Plata, Argentina; ^3^Center for Research and Development in Food Cryotechnology (CIDCA, CCT-CONICET La Plata), La Plata, Argentina

**Keywords:** Circular Economy, sustainability, fruits and vegetables, food systems, Green Deal

## Abstract

Fruits and vegetables wastes (*e.g*., peel fractions, pulps, pomace, and seeds) represent ~16% of total food waste and contribute ~6% to global greenhouse gas emissions. The diversity of the fruit-horticultural production in several developing countries and the excess of certain fruits or vegetables in the months of greatest production offer unique opportunities for adding value to these wastes (co-products). Within the scope of the Circular Economy, valorization of such wastes for the production of innovative bio-ingredients can open great market opportunities if efficiently exploited. In this context, this review deals with the current situation of wastes arising from fruits and vegetables (availability, characterization) as sources of valuable ingredients (fiber, polyphenols, pigments) suitable to be incorporated into food, pharmaceutical and cosmeceutical products. In addition, an integral and systematic approach including the sustainable technologies generally used at both lab and industrial scale for efficient extraction of bioactive compounds from fruits and vegetables wastes are addressed. Overall, this review provides a general updated overview regarding the situation of fruits and vegetables chain supplies in the post-pandemic era, offering an integrative perspective that goes beyond the recovery of fiber and phytochemicals from the previous mentioned wastes and focuses on whole processes and in their social and economic impacts.

## Introduction

Food supply chains are highly integrated and strongly dependent on global conditions (1). Therefore, economic, political threats and natural disasters may disrupt their integrity. The COVID-19 pandemic has put into evidence the lack of resilience of food supply chains, leading to economic and social crises with global consequences. The post-pandemic reactivation process requires accelerated economic growth to recover losses and reduce social suffering. In this context, when global markets are damaged or slowed down, the Circular Economy offers strategic solutions, turning wastes into sustainable resources (1). Therefore, the implementation of production management models enabling better use of local/regional resources is a key concept to afford this post-pandemic crisis.

Reactivation strategies based on Circular Economy have been strongly encouraged (2). In this regard, the United Nations have expressed their great concern about the sustainability of human activities at a worldwide level. In particular, target 12.3 of the progress report 2019 on sustainable development goals (SDG) ambitions to halve the global food waste at the retail and consumer levels and reduce food losses along production and supply chains, including post-harvest losses by 2030 (3). In this line, different international programs based in developed countries (*e.g.*, the European Green Deal and the Chinese Ecological Civilization, Green New Deal-South Korea) have addressed lateral and complementary aspects of this global commitment (2). In contrast, developing countries have the longest way to go. Indeed, as Circular Economy is a concept not yet fully incorporated, sustainable solutions need firstly to be implemented in the culture of food supply chain actors (3).

In this context, the successful implementation of recovery strategies based on Circular Economy requires a thorough analysis regarding the origin and availability of food wastes in each region in order to employ the most appropriate approach to add them value (3, 4). Their valorization appears as a strategic engine for the development of new ventures contributing to the recovery and resilience of social and natural systems in the post-pandemic era, paving the way toward the Green Deal. The greatest challenge is to encourage the culture of sustainability in the development of industrial processes (3). This review will be focused on fruits and vegetables food supply chains, which lead to large amounts of local wastes that create enormous opportunities to design strategic resilience plans for the recovery of valuable compounds. Therefore, the problematic and opportunities that fruits and vegetables food supply chains offer will be addressed. In addition, the most relevant functional ingredients that can be obtained from them will be discussed, as well as the most suitable extraction processes. The social impact of incorporating these compounds into innovative processes will be discussed together with their market perspectives.

## Problems and Opportunities in Fruit and Vegetables Supply Chains

Although both “food loss” and “food waste” contribute to unsustainable food supply chains, it is important to point out the differences. Whereas, “food loss” is mainly caused by the malfunctioning of the food production and supply system or its institutional and policy framework (*e.g*., management, technical limitations, lack of storage facilities, cold chain, proper handling practices, infrastructure, packaging and efficient marketing systems), “food waste” refers to the removal of still consumable products from the food supply chain (either by choice or after expiration of food products, as result of poor stock management or neglect) (3). Regardless the origin of sustainability problems (food losses or wastes), tackling them in efficient, sustainable and integrated ways appears as a smart strategy to both feed people and optimize the use of natural and financial resources. This approach requires the optimization of food processing procedures, streamlining supply chains and linking farmers to markets.

Fruits and vegetables lead the ranking of food wastes and losses (40–50% of their production), thus resulting in the use of non-renewable resources to obtain food products that will not be consumed [*ca*. 25% of all water used by agriculture each year (4) and 23% of all cropland, producing about 8% of annual global greenhouse gases emissions (5)]. Whereas, food loss mainly takes place at the earlier stages of the food supply chain (production, post-harvest and processing stages), food waste mostly occurs (but not exclusively) at the retail and consumer levels. Likewise, food waste is particularly problematic in developed/industrialized countries and food loss, in developing ones (6).

**Figure 1:**
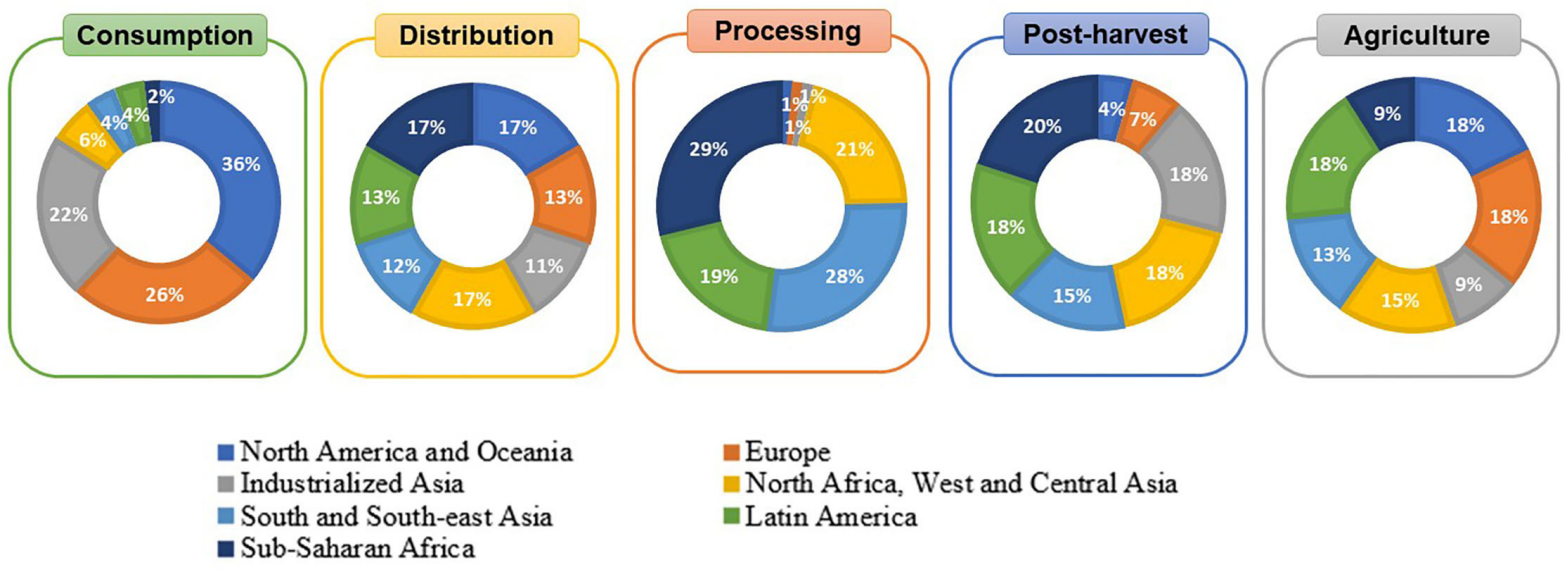
Percentage of fruits and vegetables' losses and wastes at different stages of the food supply chain in different regions. Each circle represents the total (100%) losses and wastes arising from each stage at the worldwide level. The contribution of each region (expressed in percentage) is also indicated in the Figure. Different colors represent different geographical regions. Information taken from reference (8).

The losses arising from fruits and vegetables (*e.g*., peel fractions, pulps, pomace, seeds) represent ~16% of total food waste and contribute ~6% to global greenhouse gas emissions at a worldwide level both in developed and developing countries (6, 7). They can occur at five different stages, namely *agriculture* (harvest operation and subsequent sorting and grading), *postharvest* (handling, transportation and storage after harvest and before processing), *processing, distribution* and *consumption*. In developing countries, 9 to 18% of the losses occur at agriculture and 15–20%, at postharvest stages as a result of the lack of infrastructure and inappropriate handling operations. This leads to early harvesting, technical limitations and insufficient storage (7). In contrast, in developed countries, the losses ascribed to these two first stages represent the lowest percentages, the major losses occurring at the last stages (*e.g*., retail and consumers, demanding high-quality products and rejecting products with ugly appearance) (Figure 1) (7, 9). This puts into relevance the importance of improving processing technologies for perishable products, including fruits and vegetables, and/or adding them value by applying innovation for the development of functional ingredients (10).

As fruits and vegetables are fresh products, they are altered by the advance of maturation or senescence, being discarded because they do not have the quality required for consumption. In addition, as many of them are seasonal products with a very short production and marketing cycle (2–3 months), producers mainly use their harvest for fresh commercialization at the local level. This generates high amounts of residues due to their perishability and market saturation in times of production. This perishability is partially solved by dehydrating such products. However, the losses of fruits and vegetables are still high, especially in developing countries, where innovation can play a key role to overcome this problem. The diversity of fruits and vegetables in many developing countries offers great availability of losses that can be transformed into valuable inputs for the development of innovative products (*e.g*., production of diverse sustainable biomaterials, energy, high-value products). This approach also promotes socioeconomic development by generating new products, processes, equipment, regulations, and increasing qualified jobs (11).

**Figure 2:**
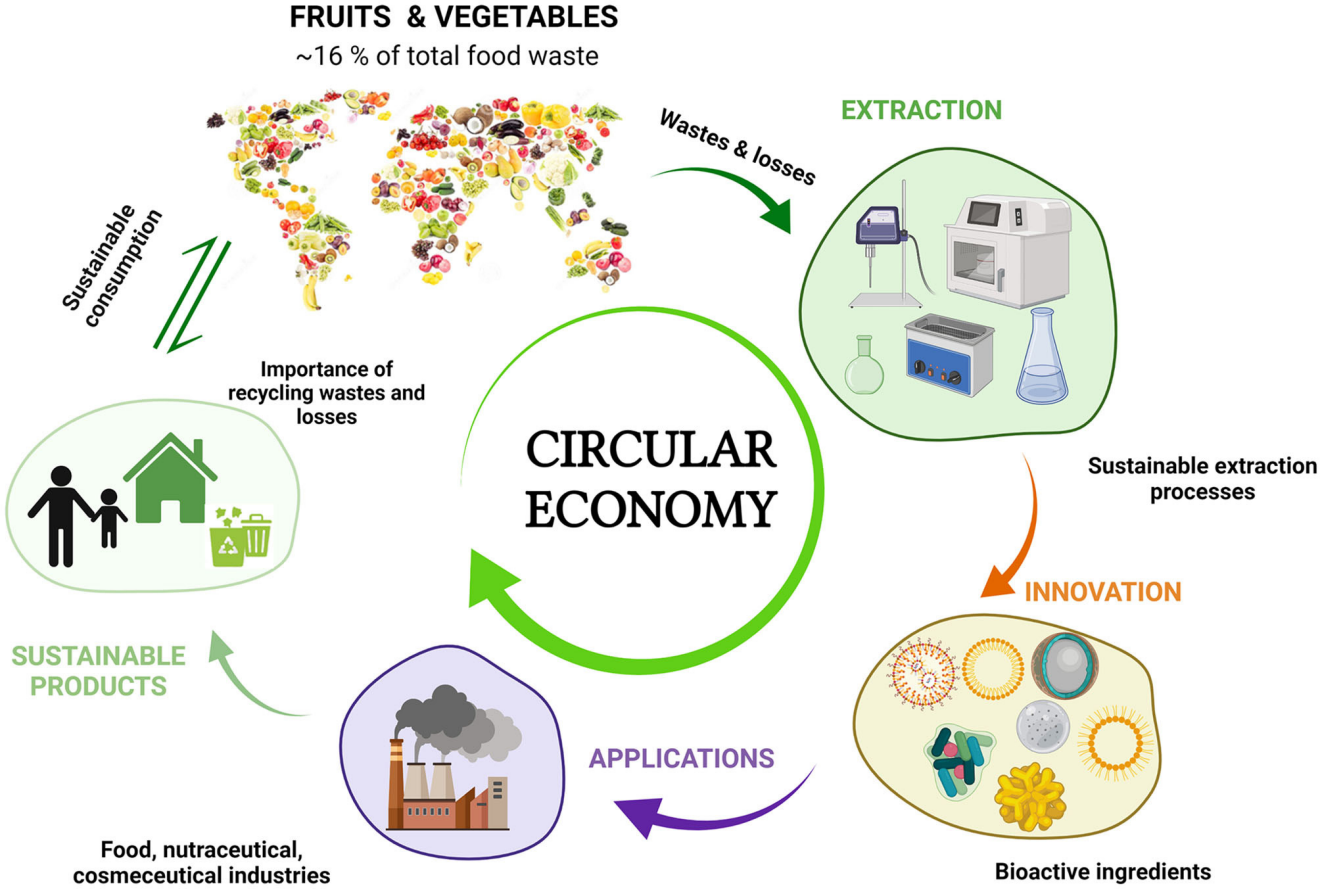
Suitability of valorization of fruits and vegetables within the scope of the Circular Economy concept.

Taking this into account, food losses resulting from the production of fruits and vegetables constitute unique starting materials for the recovery of different valuable compounds in a Circular Economy model (8, 12), thus providing responses for the post-pandemic recovery (Figure 2). In particular, peels, pulps, pomaces and seed fractions of fruit and vegetables can be suitable raw materials for the recovery of different bioactive compounds, including fiber (pectins, prebiotic oligosaccharides), polyphenols and pigments, among the most important ones. Such opportunities are specifically addressed in Functional ingredients in agro-wastes of fruits and vegetables, Sustainable extraction methods, and Social impact of incorporating bioactive compounds from regional fruits and vegetables by-products into innovative processes.

## Functional Ingredients in Agro-Wastes of Fruits and Vegetables

As mentioned in the previous section, losses and wastes in the production of fruits and vegetables (pomace, skin, seed, stem, leaves, roots, tubers, etc.) occur worldwide along all stages of the food supply chain and also, after reaching the consumers (Figure 1). The appropriate waste management should be focused on reducing the amounts of residues instead of re-using, recycling, and disposing them. Nevertheless, most of the food wastes are still landfilled, composted and incinerated for the production of biogas, leading to the emission of harmful gases that cause severe environmental problems (13).

The design of sustainable strategies to efficiently reduce fruits and vegetables wastes' production is highly required and particularly valuable considering that they are underexploited sources of nutritionally relevant bioactive compounds (*e.g*., polyphenols, pigments, dietary fiber) with potential health-related properties. Such beneficial effects include antioxidant, antimicrobial, anti-inflammatory, anti-immunomodulatory and cardioprotective activity, among others. Pomace is the main solid waste obtained in high quantities after the extraction of juices from fruits and vegetables, and mainly consists of peels, residual pulp, and seeds. Therefore, the extraction of phytochemicals, the recovery of dietary fiber, or the use of the whole pomace to functionalize and add value to food products, supplements, and cosmetic formulations are promising ways to efficiently use these fruits and vegetables by-products (losses and wastes) (Figure 2). This may contribute to food wastes reduction with additional economic and nutritional benefits (13). In the next subsections, the importance of adding value to the main bioactive compounds (polyphenols and pigments) and dietary fiber present in the losses/wastes of fruits and vegetables is deeply discussed.

### Dietary Fiber

The wastes and losses of fruits and vegetables are rich sources of total dietary fiber (Table 1), having a higher soluble fiber proportion, better insoluble/soluble fiber ratio, lower caloric content, lower phytic acid content and better functional properties than those obtained from cereal processing (the traditional source of fiber) (32). In particular, pectin is the main type of soluble fiber found in fruits and vegetables wastes. This carbohydrate was reported in apple pomace, cranberry pomace, grapes pomace and stalk, mango peel, pear pomace, date flesh, grapefruit peel, carrot pomace, whole tomato and potato, and pumpkin skin. Regarding insoluble fiber, cellulose, hemicellulose and lignin were identified in most of fruits and vegetables wastes previously mentioned (33). In addition, these polysaccharides were also found in orange pomace, peach pomace, pineapple leaves and stem, pomegranate peel, banana peel, cauliflower waste, onion skin, garlic husk and pea hulls.

**Table 1:** Dietary fiber and bioactive compounds of the main fruit and vegetables by-products.

**Fruit/ vegetable**	**Total dietary fiber (% w/w dm)**	**Soluble fiber** **(% w/w dm)**	**Insoluble fiber (% w/w dm)**	**Total phenolic content**	**Total carotenoids**	**References**
**Pomace**						
Apple	51.10	14.60	36.50	10.16 mg GAE/g	-	(14)
Blackcurrant	59.13	3.97	55.16	32 mAU min/g	-	(15)
Carrot	63.6	13.5	50.1	-	-	(16)
Chokeberry	59.50	7.04	52.46	155 mAU min/g	-	(15)
Gooseberry	56.60	7.04	49.56	10 mAU min/g	-	(15)
Redcurrant	58.08	7.00	51.08	20 mAU min/g	-	(15)
Red wine grape	51.09–56.31	0.81–1.72^a^	49.59–54.59^a^	21.4–26.7 mg GAE/g	-	(17)
Rowanberry	67.17	7.68	59.49	37 mAU min/g	-	(15)
Tomato	64.12	5.56	58.54	55.10 mg GAE/g	-	(18)
White wine grape	17.28–28.01	0.72–0.84^a^	16.44–27.29^a^	11.6–15.8 mg GAE/g		(17)
**Peel**						
Apple	47.8	5.8	42.1	28.3 mg GAE/g	-	(19)
Avocado	6.85–56.9	-	-	181.2–22,790 mg GAE/100 g	0.89–2.6 mg/100 g	(20)
Citrus	67.42	4.94	62.48	145.5 mg GAE/100 g	-	(21)
Mango	69.86	24.63	44.23	4.5 mg GAE/100 g	5,600 μg β-carotene /g	(22)
Persimmon	40.35	-	-	-	340 mg/100 g	(23)
Plantain	64.33	7.45	56.88	15.21 mg QE/g	-	(24)
Sharlyn melon	29.59	-	-	66.2–325.3 μg/g dw	76.80 g β-carotene/ 100 g	(25)
Tomato	86.15	14.33	71.82	158.10 mg GAE/100 g	3–4 mg lycopene/100 g	(26)
**Seed**						
Avocado	2.87–26.33	-	-	0.29–0.53%	-	(27)
Grapes	8.15	-	-	0.47 g/100 g	-	(28)
Papaya	7.75–8.78	5.24–5.44	2.51–3.36	34.0–91.6 mg GAE/g	-	(29, 30)
Pomegranate	-	-	-	0.29–22.61 mg/L extract	-	(31)

a *mg galacturonic acid equivalents GUAE/g DM*.

The consumption of dietary fiber is associated with several health beneficial properties, including enhancement of the digestive system, promotion of intestinal peristalsis, prevention of cardiovascular diseases, decrease of glucose absorption and control of insulin response, decrease of cholesterol levels and increase in satiety sensation (34). Additionally, dietary fiber can act as a substrate to colonic microbiota, showing a prebiotic effect (34). Moreover, several bioactive compounds present in fruits and vegetables (*e.g*., phenolics, flavonoids, carotenoids) are usually bound to dietary fiber, which reinforces their antioxidant activity. The resulting association was called ‘antioxidant dietary fiber' by Saura-Calixto (35) and was identified in numerous fruits and vegetables matrices (36). From a technological viewpoint, dietary fiber has some interesting properties, including a high-water holding capacity, swelling capacity and gel formation, which can be useful to increase the texture and viscosity of certain food products. Besides that, dietary fiber from fruits and vegetables has higher oil holding capacity than some legumes, and this property can be useful to avoid fat losses and flavors during food processing, especially in meat products (37). The health-promoting properties and functionality of dietary fiber have encouraged producers to include this ingredient in different food and nutraceutical formulations, already introduced in the market. Indeed, the main applications of dietary fiber in food industry include beverage (to enhance stability and viscosity), bakery (to improve the nutritional composition and water retention), dairy products (to increase consistency, rheology properties, texture), meat (to improve the nutritional composition, viscosity, emulsion stability) or as food additives (33). For example, Sudha et al. (14) studied the addition of apple pomace to wheat flour (5–15%) and observed that the increase of apple pomace levels leads to an increase of water absorption and resistance to extension together with a decrease of extensibility and viscosity. In addition, these authors studied the baking properties when prepared a cake with wheat flour containing apple pomace (0–30%) and observed that as the apple pomace content increases, the cake becomes harder with a decreased volume. Also, when apple pomace is incorporated at a higher concentration, the sensory quality of cakes is negatively affected (14). In this line, Chau et al. (16) reported that the water-insoluble fiber of carrot pomace has the best functional properties (swelling capacity, water- and oil-holding capacity), leading to better glucose-adsorption capacity, and amylase-inhibition activity. Similarly, apple pomace is a rich source of dietary fiber (45–60 %) and associated phytochemicals, mainly composed of insoluble fiber, namely cellulose (20.20–43.60 %), hemicelluloses (4.26–24.40 %) and lignins (15.30–23.50 %) (37). The contribution of soluble fiber to the total dietary fiber is also important, pectins (5.50–11.70 %) and gums being the predominant compounds (37).

Grape pomace is another important by-product mainly composed of seeds and skin and is obtained in large quantities from winery (~20% w/w of the total grapes), after pressing and/or fermentation processes. Similar to other fruits and vegetables by-products, the insoluble fiber is the major fraction (51.70 %w/w) contributing to the total dietary fiber of grape pomace (57.24 %w/w) and is mainly composed of cellulose, lignin and hemicellulose. Zhang et al. (38) reported that after extracting phenolic compounds of grape pomace, the insoluble fiber content significantly increases whereas the soluble fraction (*e.g*., pectins, gums, inulin, arabinoxylan, xylose and raffinose) is solubilized together with phenolic compounds.

Dietary fiber represents a large percentage in the wastes and losses of most fruits and vegetables. Incorporating it in food products not only provides a source of health benefits, but also has several technological properties. Considering the awareness of consumers to ingest nutritious products and avoid the consumption of saturated lipids or trans fatty acids, using fiber-based replacers is an increasing trend that could offer additional ways to diversify the possibilities of adding value to fiber containing losses and wastes. Besides that, considering the chemical nature of fiber (polysaccharides) and the wide range of applications of such compounds, fiber from fruits and vegetables wastes and losses certainly has enlarged application possibilities. As observed in the papers referred in the previous paragraphs and in Table 1, different approaches have been already carried out. However, there is still a long way to go, and this will not only contribute to use by-products but also to provide healthier and sustainable products to the population.

### Polyphenolic Compounds

The losses and wastes of fruits and vegetables are also rich sources of polyphenols, a large group of phytochemical compounds with important biological functions in human health. The main bioactive activity attributed to polyphenols is their antioxidant activity as these compounds can scavenge reactive oxygen species (ROS) produced in any oxidative environment which trigger degenerative and inflammatory diseases (39). However, the improvement of cardiovascular health by polyphenols has also been related to their ability to regulate transcriptional genes and modulate enzymes playing key roles in the synthesis of inflammation mediators (40). From a chemical point of view, although polyphenols include a great variety of compounds with different chemical structures, all of them have one or more hydroxyl groups (–OH) bound to at least, one aromatic ring (41). The different classes and subclasses of polyphenols are subdivided on a biosynthetic basis as follows: phenolic acids, lignans, stilbenoids, flavonoids and tannins (40). Several works have suggested that after industrial processing, polyphenols can be present in fruits and vegetables by-products, even in a higher proportion than in the edible parts (13). In this way, Li et al. (42) identified chlorogenic acid, quercetin-3-O-galactoside, quercetin-3-O-rhamnoside, and phloridzin as extractable phenolic compounds in apple pomace. These authors exposed such by-products to acid and basic hydrolysis (as single or combined procedures) to promote the release of polyphenols bound to the fruit matrix. While acid hydrolysis breaks glycosidic bonds, alkali does it for ether and ester bonds. Hence, acid hydrolysis promotes the release of significant quantities of 4-hydroxybenzoic acid and isoferulic acid, whereas the basic procedure induces the extraction of protocatechuic acid and catechin. These authors also observed that fractions with higher phenolic compounds are those obtained after basic hydrolysis, which led to higher antioxidant activity through oxygen radical absorbance capacity (ORAC) assay thus, supporting the application of extraction and hydrolysis procedures to the recovery of polyphenols from apple by-products (42). Similarly, Abbasi-Parizad et al. (43) analyzed the polyphenol composition of different agro-industrial by-products (grape pomace, spent coffee grounds, tomato pomace, and red corn cobs) and found that flavonoids (quercetin, rutin, apigenin, and naringenin) represent the 95% of total polyphenols in grape pomace, whereas gallic acid and chlorogenic acids are the main polyphenols in spent coffee grounds. Tomato pomace showed a higher concentration of cinnamic acid, p-coumaric and caffeic acids. In addition, flavonoids have a higher contribution to the polyphenolic composition (65%), represented by naringenin and naringenin chalcone (43). Red corn cob has higher quantities of chlorogenic acid and ferulic acids and, with an important contribution of flavonoids (catechin and epicatechin). These authors also observed that spent coffee grounds and grape pomace exhibit better anti-inflammatory activities than the other by-products and this was attributed to the high flavonoid contents, thus concluding that spent coffee grounds and grape pomace may have the potential to be industrially recovered (43).

Polyphenols include a great variety of chemical compounds, ranging from small molecules to highly polymerized ones. Fruits and vegetables (and also their wastes and losses) are one of the richest sources of natural polyphenols. Considering the large availability of diverse fruits and vegetables worldwide, and the large percentage that is lost at the different stages of the production (Figure 1), there exists great amounts of underexploited polyphenols. Although they have been correctly identified, their exploitation requires appropriate and sustainable extraction methods, with costs compatible with the fact of using agro-industrial by-products (it would be non-sense the use of expensive extraction methods even when they were sustainable). This highlights the important role of innovation for a better exploitation of natural polyphenols from fruits and vegetables food supplies.

### Pigments

The global dyes and pigments market size is expected to reach USD 51.7 billion by 2028 and is projected to expand at a compound annual growth rate (CAGR) of 5.1% from 2021 to 2028 (44). However, the synthetic pigments traditionally employed in the food, nutraceutical and cosmeceutical industries are more and more controversial not only because of their unsustainable production (chemical synthesis) but also because, although accepted, their consumption is less safe than that of pigments arising from other sources (45).

This situation encouraged producers to obtain pigments from renewable resources. Fruits and vegetables' wastes and losses (seeds, pomace, peels) are rich and safer sources of natural pigments. Therefore, their valorization is an interesting strategy to meet the demands of natural pigments production at the industrial level. Their intense colors provide natural pigments to be used in the formulation of different products, such as juices, candies, chocolates, bakery and confectionery, jams and jellies, instant drink powders, sauces, ice-creams, among others. What is more, the health beneficial effects of such pigments (*e.g*., antioxidant, anti-inflammatory, anticancer, antimicrobial, cardioprotective, antithrombotic) offers an additional advantage, providing not only colorants but also functional ingredients (9). The challenge that industrials have currently to face is to find sustainable approaches for their production, including green extraction and processing technologies, encapsulation techniques and retaining their health beneficial effects along processing.

**Figure 3:**
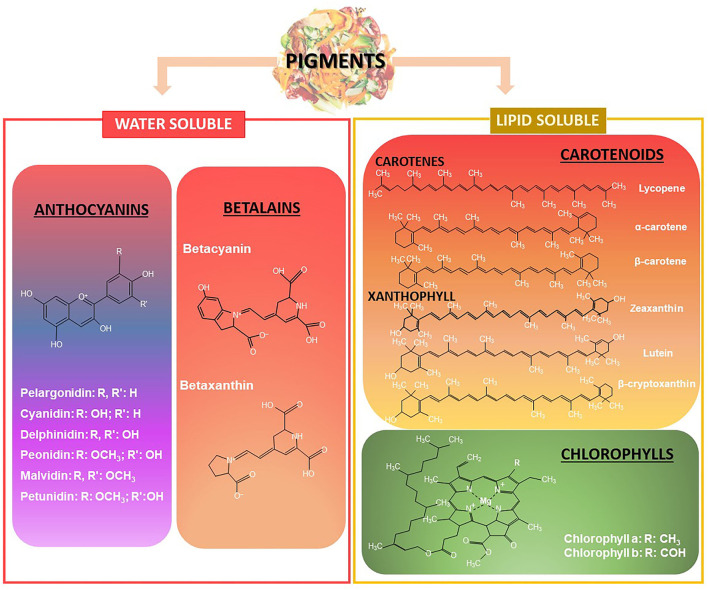
Representative chemical structures from the most common types of natural pigments present in food wastes and losses.

From a chemical and structural viewpoint, natural pigments can be classified into four major groups, namely anthocyanins, betalains, chlorophyll and carotenoids. Anthocyanins (red, blue, purple) and betalains (red) are water soluble pigments whereas carotenoids (yellow, orange, red) and chlorophylls (green) are mostly hydrophobic (Figure 3). These characteristics determine the extraction and stabilization methods employed for their sustainable valorization.

Anthocyanins represent the largest group of polyphenolic pigments, being water-soluble and non-toxic. Anthocyanins are mostly in the form of glycoside or acylated anthocyanins whereas anthocyanidins are known as the aglycones, grouped into 3-hydroxyanthocyanidins, 3-deoxyanthocyanidins, and O-methylated anthocyanidins (46). The major plant-based occurring anthocyanins are cyanidin, pelargonidin, delphinidin, peonidin, petunidin and malvidin. The structural differences arising from the number and position of hydroxyl groups are responsible for the different colors of anthocyanins (47). The wine and juice industries offer large amounts of skins and pomaces suitable for the extraction of anthocyanins to be employed in various food applications. In fact, anthocyanins can be extracted from grapes (*Vitis vinifera*) skin and pomace, apple (*Malus domestica*) peel, banana (*Musa* sp.) peel, eggplants (*Solanum melongena*) peel, blackcurrant (*Ribes nigrum*) wastes, blackberries (*Rubus* sp.) by-products, coffee (*Coffea* sp.; *C. arabica, C. robusta*) exocarp, cherries (*Prunus cerasus*) skin and pomace, jabaticaba (*Plinia cauliflora*) peel and pomace, acerola (*Malpighia emarginata*) residues, purple potatoes (*Solanum tuberosum*) peels as the main sources (48).

Betalains are classified into betacyanins and betaxanthins (Figure 3) and can be glycosylated or glycosylated with ferulic, p-coumaric, or 3-hydroxy-3-methylglutaric acids. They can be extracted from red pitaya (*Hylocereus polyrhizus*) and dragon fruit (*Hylocereus undatus*) peels, prickly pear (*Opuntia joconoste*) pericarp, ulluco (*Ullucus tuberosus*) peel and beetroot (*Beta vulgaris*) peel and pomace.

Chlorophylls are lipophilic compounds composed of a porphyrin head group bound to hydrocarbon tails (phytol group) (Figure 3). There exist two types of chlorophylls, chlorophyll a and chlorophyll b, differing in the presence of a methyl or an aldehyde group at C7, respectively. Such structural difference is responsible for the blue-green color of chlorophyll a, and the yellow-green one of chlorophyll b (49). They are ubiquitous compounds in vegetables and their discards, occurring in cucumber (*Cucumis sativus*) and watermelon (*Citrullus lanatus*) peels, spinach (*Spinacia oleracea*) by-products, as some examples (48).

Carotenoids is a large group of pigments, including carotenes (α-carotene, β-carotene and lycopene) and xanthophylls (lutein, zeaxanthin and β-cryptoxanthin). The structural difference between these two groups is the presence of hydroxyl groups (Figure 3), which imparts a less hydrophobic character to the latter group, also conditioning the extraction methods and incorporation into industrial products.

Carotenoids are abundant in tomato (*Solanum lycopersicum*) skin and pomace, gac (*Momordica cochinchinensis*) peel, cashew apple (*Anacardium occidentale*) by-products, pomegranate (*Punica granatum*) peel, acerola (*Malpighia emarginata*) residue. β-carotene occurs in orange (*Citrus X sinensis*), pomegranate (*Punica granatum*) and mango (*Mangifera indica*) peels, carrot (*Daucus carota*) peel, tomato (*Solanum lycopersicum*) peels and by-products, pumpkin (*Cucurbita pepo*) seeds and peels, sweet potato (*Ipomoea batatas*), apricot (*Prunus armeniaca*) and peach (*Prunus persica*) peels, and pepper (*Piper nigrum*) wastes. Lutein is present in tomato (*Solanum lycopersicum*) peel, spinach (*Spinacia oleracea*) by-products, paprika (*Capsicum annuum*) leaves, sweet potato (*Ipomoea batatatas*), apricot (*Prunus ameniaca*), pumpkin (*Cucurbita pepo*), peach (*Prunus persica*) peels and pepper (*Piper nigrum*) wastes. Lycopene is the main pigment of tomato (*Solanum lycopersicum*) skins and by-products, also being present in sweet potatoes (*Ipomoea batatas*), apricot (*Prunus ameniaca*), pumpkin (*Cucurbita pepo*), peach (*Prunus persica*) peels and pepper (*Piper nigrum*) wastes (48).

The variety of vivid colors from fruits and vegetables is certainly one of the most attractive characteristics. Such colors are also present in most of their losses and wastes. Considering that colored compounds present in fruits and vegetables are not only valuable sources of natural and stable colorants, but they are also sources of functional ingredients (*e.g*., antioxidants), their advantages over synthetic colorants are clear. There exists an increasing industrial interest in replacing synthetic with natural colorants and for this reason, the great variety of natural colors present in fruits and vegetables wastes and losses have several niches to be exploited (*e.g*., food, nutraceutical, cosmeceutical industries), contributing both to provide healthier products to the population and to obtain sustainable products.

## Sustainable Extraction Methods

Different researchers' worldwide have focused their work on the development of innovative approaches to valorize losses and wastes from fruits and vegetables as suitable sources of bioactive compounds. The great stimulus of funding institutions to enhance such research lines has also contributed to the large number of papers published in the last 5 years (50). However, although by-products provide cost-effective sources of inputs, most of these efforts have been employed at the laboratory level, without a real transfer to industrial processes. The main reasons for this include the lack of standardized protocols, making very difficult the comparison among laboratories, and also slowing-down of the laboratory-industry transition.

### Fiber Extraction

Fiber concentrates can be obtained using diverse methods, and include extraction processes, enzymatic pre-treatments followed by freeze-drying or spray-drying treatments, leading to the obtaining of powders. Although these methods enable the production of fiber concentrates, they are not always sustainable from an economic viewpoint. Therefore, dehydration processes of fruits and vegetable by-products leading to a decrease in their high content of water can be economically more feasible, conducting to the transformation of by-products into flours. The processes are generally mild and also concerned to retain bioactive compounds (*e.g*., antioxidants, vitamins) that might be lost in more drastic conditions. The obtaining of flours includes a stabilization step, consisting in heat treatments leading to inactivate microorganisms or enzymes and ensure food safety, as well as biological and chemical stability. Then, the drying step aims at reducing the moisture to values below 10 g/100 g. Convective dryers, circulating air ovens, air-jet, fluidized-bed dryers or microwave ovens are the equipment mostly employed at an industrial level (51). Finally, a grinding process, leading to modify technological properties by reducing the particle size to *ca*. 500 μm, which also determines the hydration properties of the powders (the lower the particle size, the higher the water holding capacity of fiber). For particles with a size lower than 500 μm, the hydration properties can worsen as a result of changes in the soluble to insoluble dietary fiber ratio though their antioxidant activity can be enhanced (52). Storage conditions of these products require deeper insights as they have been scarcely investigated (53).

### Extraction of Polyphenols and Pigments

**Figure 4:**
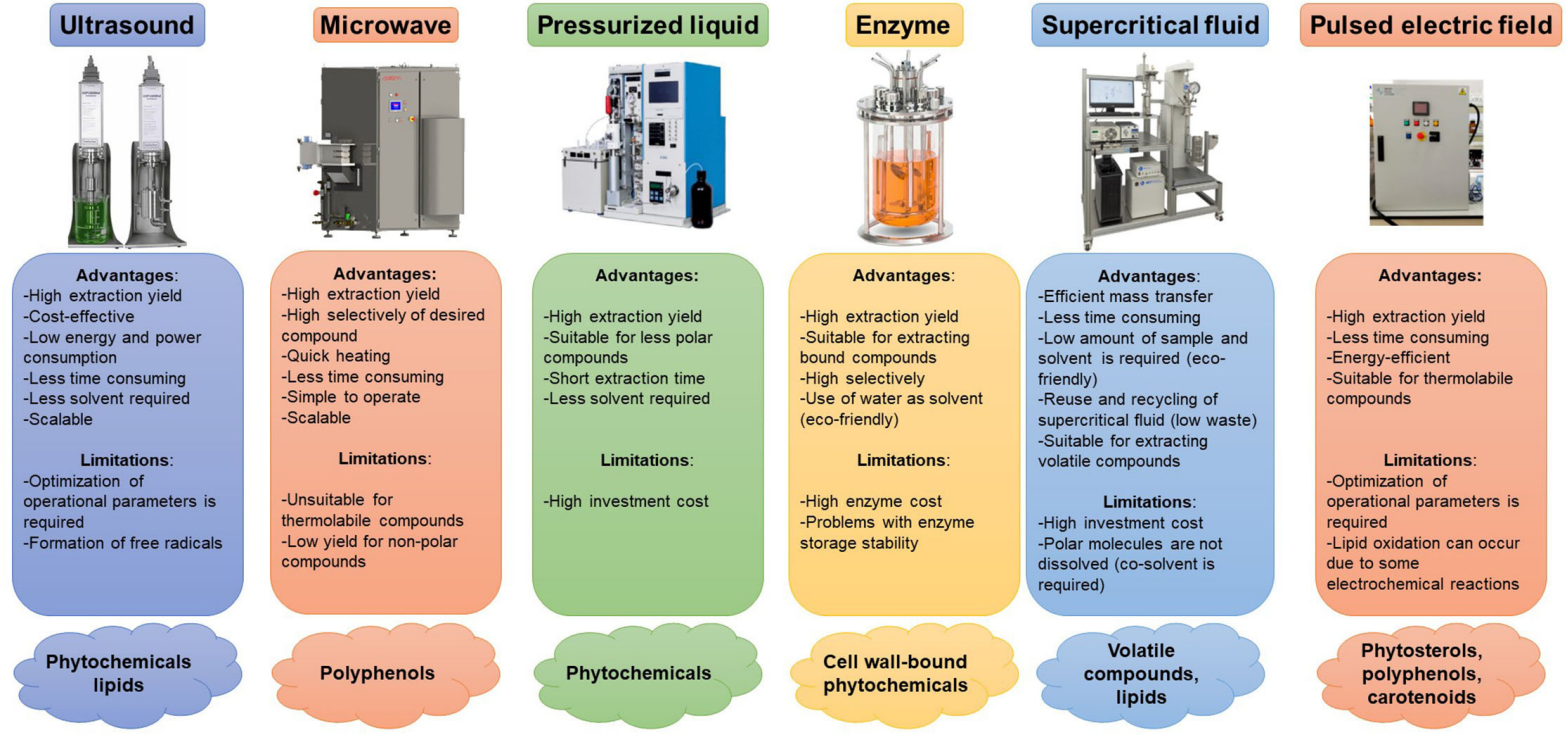
Industrial equipment for the extraction of bioactive compounds from fruits and vegetables' wastes and losses.

The industrial production of polyphenols and pigments from fruits and vegetables by-products needs suitable extraction processes. Such processes involve preliminary treatments to disrupt cell walls and other barriers of plant cells and facilitate the release of bioactive compounds. Such procedures can be classified into different categories, namely Atmospheric liquid extraction, Pressurized liquid extraction methods, Enzyme-assisted extraction, Supercritical fluid extraction and Pulsed electric field assisted extraction (Figure 4) (54). These categories basically differ in the mechanism of plant cells disruption and the operating conditions, such as temperature, pressure and solvents (55). Then, the conditions and solvents used in the extraction steps depend on the hydrophobic or hydrophilic character of the compounds.

#### Atmospheric Liquid Extraction Methods

Among the atmospheric liquid extraction, microwave and ultrasound-assisted extractions are the most employed. Ultrasonic waves generate small cavitation bubbles around the cells that, when collapsing, emit shock waves that break cell walls. This allows better penetration of solvents and increases the extraction yield of bioactive compounds, including polyphenols and hydrophobic and hydrophilic pigments (56). Ultrasound-assisted extraction coupled with probes and bath systems has been extensively used at an industrial scale. The system selection depends on the matrix, application, efficiency, and potential required (57). The companies Hielscher Ultrasonic (Germany) (https://www.hielscher.com) and REUS (France) (https://www.etsreus.com) have been developing large-scale ultrasound extraction devices for several applications in different fields (foods, beverages, pharmaceutical, cosmetics, chemistry, etc). Hielscher offers a wide variety of ultrasound systems using powerful probes (from 500 to 16,000 W) for industrial applications (58). In addition, REUS company commercializes reactors (capacity of 500–1000 L) coupled with diverse pumps, which enable continuous fill of the ultrasonic bath, maintaining the mixture stirred, also allowing its emptying once the process is finished (57).

In turn, microwave-assisted extraction is based on the interaction of electromagnetic energy in the microwave region (0.3–300 GHz) with matter. This energy induces rapid heating of the polar components of the cell, facilitating the release of bioactive compounds toward the extraction solvent, thus improving the extraction yield (58). SAIREM company (France) (https://www.sairem.com) has a leader position in developing industrial microwave equipment for thermal processing (including compounds' extraction) and plasma generation. LABOTRON series includes a wide range of integrated reactor and microwave transmission systems specially designed to perform microwave-assisted extraction or chemical synthesis in continuous flow or batch mode process. The microwave energy in LABOTRON is transmitted to the reaction via an internal transmission line (INTLI), which was patented by this company (59). The innovative INTLI technology allows a very high density of activation energy transmitted directly into the reaction medium while maintaining an efficient external cooling (http://www.testbourne.com/sairem-microwave-equipment-chemistry-the-intli-technology). The commercialized microwave generators have frequencies of 915 MHz and 2,450 MHz and offer a wide range of power, from a few watts up to 100 kW. In turn, LABOTRON can operate in a batch reactor whose volume ranges from 1.5 to 20 L or in continuous flow reaction operating at few mL/min to several L/h (59).

#### Pressurized Liquid Extraction Methods

Pressurized liquid extraction methods refer to an “accelerated solvent extraction” because using high pressure accelerates the extraction processes. Such conditions improve cell permeability, intermolecular physical interactions and penetration of the extraction solvents, thus facilitating the release of bioactive compounds (60). The release of bioactive compounds can be also accelerated by increasing the extraction temperatures, which decreases the viscosity of the solvents and facilitates their diffusion into the vegetables' matrices. The current automatization of equipment allows precise control of pressure, temperature, and time of extraction, with the possibility of simultaneously extracting bioactive compounds from large number of samples (55). As far as we know, pressurized liquid extraction equipment has not been commercialized at an industrial scale until now. However, extensive research has been carried out in simulating the economic feasibility of the large-scale application of pressurized liquid extraction. For instance, Santos et al. (61) simulated the economic feasibility of applying the pressurized liquid extraction process (batch mode; reactor capacity: 3000 L; operating 24/7 during 330 d) to extract anthocyanins and phenolic compounds from jabuticaba (*Myrciaria cauliflora*) skins using ethanol as solvent. These authors observed that the cost of manufacturing when applied the pressurized liquid extraction procedure under the optimal operation conditions (5 MPa, 553 K and 9 min of static extraction time) was 40-fold lower than values reported for conventional extraction (low pressure, room temperature, 2 h), suggesting that pressurized liquid extraction could be an economically viable technique to extract bioactive compounds at large scale (61).

#### Enzyme-Assisted Extraction

Using hydrolytic enzymes is another sustainable extraction method, enabling the disintegration of cellular structures, allowing a better release of the intracellular content and penetration of solvents, thus increasing the extraction yields (62). Enzymatic assisted methods have a great potential for extracting bioactive compounds from fruit and vegetables by-products because they combine high extraction yields with sustainability and quickness. Cellulases and pectinases are the most widely employed enzymes, the former being more efficient in matrices with high contents of polysaccharides (*e.g*., celluloses or hemicelluloses). On the contrary, when bioactive compounds are extracted from peels or seeds, pectinases are a better option (60). Several enzyme formulations are currently commercialized in the food industry for olive oil production (to improve the extraction yield, stability, nutritional quality, etc.), fruit and vegetables juices (to improve clarification and stabilization processes), and wine (to improve organoleptic attributes, stability and facilitate clarification and filtration procedures (63). In addition, the use of enzymes in the industry combined with other extraction techniques (*e.g*., supercritical fluids extraction) to improve extraction of bioactive compounds is a promising research field. Several authors have studied the use of enzymes as pretreatment for degrading cell wall plant tissues which may aid the solvent penetration and bioactive compound dissolution in the next extraction step (64). However, these approaches have only been performed at lab scale without studying the economic feasibility that includes the enzyme cost and initial investment to scale up the extraction procedure for possible industrial production.

#### Supercritical Fluid Extraction

Supercritical fluids extraction is an increasingly employed method for obtaining bioactive compounds. A supercritical fluid is any compound that is under conditions of pressure and temperature above its critical point. These characteristics provide them simultaneously with both properties from gasses (effusion) and from liquids (solvent). As above the critical conditions, small changes in pressure and temperature produce large changes in density, supercritical fluids are characterized by having a wide range of densities. Due to their liquid behavior, they facilitate the dissolution of solutes, and because of their gas behavior, they can be easily separated from the matrix. This results in the obtaining of highly concentrated extracts in a fast and easy way without traces of toxic organic solvents in the final product, also with the possibility of recycling of them (55). These characteristics outcome in sustainable, efficient and selective extraction processes, especially recommended for thermolabile compounds, including polyphenols and pigments. The great potential of supercritical fluids extraction has led technologists and engineers to invest great efforts toward automation of the processes, by online coupling sample preparation with analytical techniques (*e.g*., liquid and gas chromatography, supercritical fluid chromatography), offering the possibility of developing more efficient and fast processes (55). However, high manufacturing cost and initial investment represent the main challenges when supercritical fluids extraction is tended to scale up and thus, economic aspects should be considered.

#### Pulsed Electric Field Assisted Extraction

Pulsed electric field technology is a non-thermal methodology involving the application of repetitive high voltage pulses (in the range of μs). Such conditions promote the formation of pores on cell membranes, leading to the permeabilization of cellular tissues (65), facilitating the extractability of intracellular compounds, polyphenols and pigments (66). The main advantages of pulsed electric field extractions include their high extraction yields, no-thermal treatments and low energy usage. Although the instrumentation is expensive, the use of pulsed electric fields in the food industry has been increasing in the last years since pulsed electric field-treated foods have shown acceptable maintenance of nutritional characteristics together with the improving of microbiological quality.

The food industry is currently facing the challenge of applying sustainable approaches to recover bioactive compounds from fruits and vegetables by-products according to the Circular Economy concept. The selection of the extraction method is critical as the chemical and physical properties of bioactive compounds, including structure, polarity, location, and other characteristics strongly determine the extraction methods and the conditions to be applied. Such decisions also require the consideration of the environmental impact of processes. The selection of sustainable processes should be associated with the selection of green extraction processes and green solvents, as it would not make sense the use of not sustainable processes for the valorization of wastes and losses. However, economic aspects should not be neglected considering that some of the green extraction processes are not the most economically accessible. In this regard, life cycle assessment is a useful methodological framework to objectively, methodically, systematically and scientifically analyze the different potential environmental impacts associated with each product, process or activity, identifying and quantifying both the use of matter and energy (inputs) and the emissions to the environment (outputs) (66). LCA is an environmental management tool widely used for decision-making in selection, analysis, design and optimization processes in order to identify the most sustainable technologies both in terms of energetic consumption and in terms of economic impact (67, 68).

## Social Impact of Incorporating Bioactive Compounds From Regional Fruits And Vegetables by-Products Into Innovative Processes

Incorporating bioactive compounds (fiber, polyphenols, pigments) obtained from local fruits and vegetables by-products using sustainable processes offers not only high added value inputs for the formulation of food, nutraceutical and cosmeceutical products but is also becoming a requirement. Likewise, it stimulates the development of local industries, also contributing to decrease the carbon footprint associated with transportation.

Although the environmental concern about industrial processes dates back several years, the pandemic situation has put it into evidence, as restrictions in transportation resulted in serious problems in the supply chains in several countries. In this sense, the pandemic has reinforced the importance of producing foods and other related products at a local level using local resources, that is, strengthening the importance of more sustainable supply chains. Considering the increasing awareness of consumers about sustainable products and the increasing regulatory pressures, environmentally business commitment all along the supply chain increases the marketing perspectives of the final products, also providing suitable solutions to problems that consumers and industrials are facing in the post-pandemic era (69).

Creating integrative value chains with the appropriate business models to produce functional ingredients sustainably obtained from residual food losses appears as a smart strategy to supply affordable, healthy and natural bioactive ingredients, suitable for the formulation of functional foods, dietary supplements and also cosmetics, also contributing to overcome the economic problems associated to the pandemic.

This approach is additionally important for the creation of new job opportunities in the bio-based sector, particularly the rural, coastal and/or urban areas, also contributing to the diversification of rural economic activities, and to the valorization of locally generated losses and wastes. These new jobs involve the waste management (*e.g*., pre-treatment of residues and logistics required for the storage and transportation to the enterprises devoted to add them value), the process engineering (*e.g*., upcycling industrial plants), thus leading to the creation of both direct and indirect works at a local level. In addition, it must not be forgotten that the wide spectrum of applications of valuable compounds extracted from wastes and losses is a potential source of business expansion at an international level, thus reactivating economies in the post-pandemic era. Furthermore, the engineering of innovative products for the functional foods and cosmetic markets requires highly qualified professionals, so that this contributes to the generation of new high-tech jobs at a local level. In summary, valorizing fruits and vegetables by-products will not only contribute for environmentally sustainable practices but also create dynamic and competitive regional economies, especially in developing countries and rural environments, lowering production and transportation costs.

## Market Demands and Perspectives

The importance of implementing socially responsible strategies for producing industrial products is increasing, with great pressure for employing socially responsible operations “channeled through the supply chain” (70). At a regulation level, international organisms are strongly encouraging sustainable practices. In this sense, the European Green Deal ambitions that by 2050, all member states will have full Circular Economies, that is, achieve net-zero emissions. Therefore, companies are working hard to achieve this goal.

The COVID-19 pandemic has demonstrated that sustainability is much more than tackling environmental risks, also emphasizing the consequences of the climate and malnutrition emergency. Sustainability is an integrative process in which environmentally friendly products are the result of whole processes involving the whole chain supply. It concerns the creation of resilient infrastructure, and that is why Environmental, Social and Governance aspects are pillars for constructing resilient companies to pave the way for overcoming environmental risks. Likewise, these aspects are meticulously searched by investors before making the decision of investing in a given company.

Environmental aspects concern how companies use energy and manage their environmental impact. Social aspects are related to the way enterprises foster their people and culture (*e.g*., inclusivity, diversity), as it will have an expanding effect on the society at large. In turn, Governance concerns transparency and industry best practices, and dialogue with regulators, as well as having internal systems of controls, practices, and procedures to govern and take effective decisions. In summary, implementing Environmental, Social and Governance issues is, in practice, translated not only into a reduction of the carbon footprint but also into a higher valuation of companies. For this reason, efforts to maximize the bottom line and the pursuit of the public interest are complementary, and companies are already experiencing the financial consequences of failing to act on sustainability (*e.g.*, carbon taxes; integration of Environmental, Social and Governance aspects into the funding criteria of financial and banking sectors). To avoid poor lending conditions and exclusion from capital markets, stakeholders must develop robust sustainability and Environmental, Social and Governance strategies.

The rising concerns over food waste and sustainability are already strongly impacting the global food waste management market size. In fact, this market was estimated at 34.22 billion USD in 2019 and is expected to expand at a CAGR of 5.4% from 2020 to 2027 (71). This topic, together with the stringent regulations of governments and food management bodies, have strongly contributed to the market growth.

On the other hand, the global market for functional food ingredients was worth 20.35 billion USD in 2016, and is likely to reach a value of 32.40 billion USD by the end of 2024. According to that report, the market is predicted to register a 6.0% CAGR (2016–2024) (72). USA and Canada have led the global food ingredients market in 2017 with ~36% of the global market followed by Europe and Asia-Pacific.

The global functional foods' market was valued at 153,600 million USD in 2018 and is expected to reach 260,400 million USD by 2025, growing at a CAGR of 6.8% during 2019–2025. Factors that are promoting consumer interest in functional foods have increased health care costs, aging, and growth in interest in attaining wellness through diet. Asia Pacific accounted for over 45% of the global market in 2017 and it is expected that they will maintain a lead position over the forecast period (72).

Regarding the nutraceutical market, the global was estimated at 115.06 billion USD in 2018 and it is expected to expand at a CAGR of 7.8% during 2021–2028 (73). Rising health concerns along with changing lifestyles and diets have stimulated the demand for nutraceuticals. In general, consumers' attitude is very positive regarding dietary supplements with added health and wellness benefits.

In turn, as for the cosmeceutical industry, the natural and organic cosmetics market is expected to reach revenues worth 25,107.7 million USD, expanding at a CAGR of 9.60 % during 2021–2025 (74). The market growth for Europe is expected to register a 9.71% CAGR during the forecast period. Its worth by 2023 is expected to be worth 8155.0 million USD. The growth of the skincare products segment in the global natural and organic skincare products market is driven primarily by increasing demand for clean label products, coupled with increasing number of health-awareness consumers globally.

Because of the prevalence of lifestyle diseases and the growing geriatric population, worldwide consumers are becoming health-conscious. They are now shifting from chemically derived products to preventive healthcare items like nutraceuticals that contain safer, natural and healthier ingredients, millennials being the generation with the highest health awareness. In particular, the awareness of consumers for reducing the contents of sugars and saturated lipids in their diets represents a great opportunity for the market of valuable and natural, offering new business opportunities.

When considering the growth of food, nutraceutical and cosmetics markets together with the availability of fruits and vegetables by-products, which in turn represent a very promising market (71), rich in valuable ingredients, it can be concluded that the post-pandemic era offers great opportunities for the development of sustainable businesses where innovation is a key actor. The success of such businesses needs adequate communication between all stakeholders, including local fruits and vegetables producers, researchers, technologists and industrials. The need of this intersectoral interaction is expected to have been a lesson learned from the COVID-19 pandemic.

## Conclusion

In the last years, Circular Economy has become a real paradigm in many different fields of knowledge. The translation of a lineal economy, based on extracting, consuming and disposing resources that negatively impact on the availability of natural resources worldwide, into a circular one, in which resources remain usable as much time as possible also avoiding the unnecessary generation of waste, has demonstrated enormous advantages both for a better use of natural resources and also to avoid or minimize the environmental impact of industrial processes. Awareness of the scarcity and depletion of non-renewable resources, strongly supported by sounded scientific evidence, international organisms (*e.g*., United Nations, European Commission) have demonstrated their concern about sustainability. Taking into account that people all over the world are inhabitants of the same planet, the promotion of sustainable practices concerns all of us.

Considering that about 40–50% of the worldwide production of fruits and vegetables is discarded either as waste or as loss, adding them value offers great opportunities for the development of Circular Economy at different levels of the production process. This integrative vision that goes beyond the valorization and extraction of phytochemicals from fruits and vegetables and focusing on whole processes as well as their social and economic impacts, not forgetting the current behavior of the market let this review provide a general updated overview regarding the situation of fruits and vegetables chain supplies in the post-pandemic era.

Going in that direction requires an integrative approach involving many different actors, including primary producers, which must incorporate sustainable practices in agriculture, post-harvest and processing, sustainability in the distribution step, and aware consumers at the consumption stage. Moreover, as losses and wastes from fruits and vegetables are rich in many bioactive compounds suitable to be incorporated into food, nutraceutical and cosmeceutical products, they can open great market opportunities if smartly exploited. In this regard, innovation plays key roles both in the development of sustainable extraction methods and in the formulation of novel healthy products. The CAGR values of functional foods, nutraceuticals and cosmeceutical markets strongly support the great opportunities of fruit and vegetables chain supplies, as rich and cost-effective sources of bioactive compounds for these markets. Adding them value requires the development of sustainable processes, also providing great opportunities for innovation, which are so necessary for the accelerated economic growth required to overcome the economic and social problems associated to the SARS-Cov-2 pandemic.

## Author Contributions

LC: conceptualization, discussion and writing of problems and opportunities in fruit and vegetables supply chains, Table 1 and Figures 1–4. AG-Z: conceptualization, discussion and writing of sections introduction, functional ingredients in agro-wastes of fruits and vegetables, sustainable extraction methods, social impact of incorporating bioactive compounds from regional fruits and vegetables by-products in to innovative processes, and market demands and perpectives. Both authors have approved the final version of the manuscript.

## Funding

This work has received funding from the Argentinean Agency for the Scientific and Technological Promotion [PICT start-up (2016)/4808 and PICT (2017)/1344]. LC and AG-Z are members of the research career of the Argentinean Research Council (CONICET).

## Conflict of Interest

The authors declare that the research was conducted in the absence of any commercial or financial relationships that could be construed as a potential conflict of interest.

## Publisher's Note

All claims expressed in this article are solely those of the authors and do not necessarily represent those of their affiliated organizations, or those of the publisher, the editors and the reviewers. Any product that may be evaluated in this article, or claim that may be made by its manufacturer, is not guaranteed or endorsed by the publisher.
